# A mixed methods feasibility study of the Kusamala Program at a nutritional rehabilitation unit in Malawi

**DOI:** 10.1186/s40814-018-0347-8

**Published:** 2018-09-24

**Authors:** Allison I Daniel, Meta van den Heuvel, Melissa Gladstone, Mike Bwanali, Wieger Voskuijl, Celine Bourdon, Isabel Potani, Sara Fernandes, Jenala Njirammadzi, Robert H J Bandsma

**Affiliations:** 1 Centre for Global Child Health, Hospital for Sick Children, Toronto, Ontario Canada; 2 Division of Gastroenterology, Hepatology and Nutrition, Hospital for Sick Children, Toronto, Ontario Canada; 3 Department of Nutritional Sciences, Faculty of Medicine, University of Toronto, Toronto, Ontario Canada; 4 Division of Paediatric Medicine, Hospital for Sick Children, Toronto, Ontario Canada; 5 Department of Paediatrics, Faculty of Medicine, University of Toronto, Toronto, Ontario Canada; 6 Department of Women’s and Children’s Health, Institute of Translational Medicine, University of Liverpool, Liverpool, UK; 7 Moyo Nutritional Rehabilitation and Research Unit, Queen Elizabeth Central Hospital, Blantyre, Malawi; 8 Global Child Health Group, Emma Children’s Hospital, Academic Medical Centre, University of Amsterdam, Amsterdam, The Netherlands; 9 Department of Paediatrics and Child Health, College of Medicine, University of Malawi, Blantyre, Malawi; 10 The Childhood Acute Illness & Nutrition Network (CHAIN), Nairobi, Kenya; 11 Department of Biomedical Sciences, College of Medicine, University of Malawi, Blantyre, Malawi

**Keywords:** Severe acute malnutrition, Child development, Nutrition, WASH, Psychosocial stimulation, Integrated interventions

## Abstract

**Background:**

Children admitted to nutritional rehabilitation units (NRUs) for inpatient treatment of severe acute malnutrition (SAM) are at high risk of poor developmental and nutritional outcomes. The Kusamala Program is an interactive hospital-based counseling program for primary caregivers of children with SAM that integrates three modules: nutrition and feeding; water, sanitation, and hygiene (WASH); and psychosocial stimulation. This mixed methods feasibility study aimed to evaluate the implementation of the Kusamala Program in an NRU setting and developmental outcomes in children with SAM 6 months after inpatient treatment.

**Methods:**

An internal pilot trial including the first 30 children and their primary caregivers enrolled to a cluster-randomized controlled trial of the Kusamala Program was performed. Barriers and enablers were identified in a qualitative study with a focus group discussion (FGD) and in-depth interviews (IDIs) with 12 hospital staff members, including five NRU nurses who deliver the Kusamala Program.

**Results:**

Results demonstrated high participant engagement (100%) and adherence (87%) of primary caregivers to the Kusamala Program. Potential barriers to implementation identified through the qualitative study were caregivers’ perceived value of the program, prioritization of other ward activities, and shortages of staff. On the other hand, enablers to implementation were engaging other staff members, motivation and work ethic, and refresher training.

**Conclusions:**

This mixed methods study demonstrates the feasibility of implementing the Kusamala Program in a real NRU setting. The full cluster-randomized controlled trial will be completed to evaluate the effectiveness of the Kusamala Program.

**Trial registration:**

ClinicalTrials.gov, NCT03072433. Registered on 7 March 2017—retrospectively registered

**Electronic supplementary material:**

The online version of this article (10.1186/s40814-018-0347-8) contains supplementary material, which is available to authorized users.

## Background

An estimated 250 million children in low- and middle-income countries around the world are unlikely to reach their developmental potential in terms of cognitive, language, socioemotional, and motor function [1–3]. There is an urgent need to implement evidence-based programs addressing child development and to integrate these programs across sectors [4–7]. Interventions that combine various modules aimed at improving development, nutritional status, and health are likely to be most effective, yet implementation of such programs has been inconsistent and isolated [4–6, 8].

Important contributors to poor child development include malnutrition itself, acute illnesses and infection, and inadequate psychosocial stimulation [3]. Therefore, some of the most susceptible children to impaired development are those with severe acute malnutrition (SAM), characterized by severe wasting or bilateral pitting edema, with complications such as diarrhea, pneumonia, malaria, or loss of appetite [9, 10]. The current Community-Based Management for Acute Malnutrition (CMAM) guidelines indicate that children with SAM and complications should be admitted for inpatient treatment at nutritional rehabilitation units (NRUs) [9, 11, 12]. While admitted to NRUs, children with SAM first are stabilized and clinical complications managed [11, 13]. Subsequently, they are nutritionally rehabilitated by providing high energy and protein feeds [11, 13].

Malawi is one of 34 countries contributing to all but 10% of the global prevalence of malnutrition [14]. Anthropometric data from the 2015–2016 Malawi Demographic and Health Survey shows that 37% of children are stunted and 3% are moderately or severely wasted [15]. The CMAM approach was adopted at the national level in 2006 in Malawi until all 28 districts in the country implemented CMAM programs by 2010 [16].

A cross-sectional study done at an NRU in Malawi known as the Moyo NRU used the Malawi Developmental Assessment Tool (MDAT) to evaluate development in children at discharge from inpatient treatment for SAM [17, 18]. These children had low developmental *z*-scores compared to a reference population of Malawian children [17, 18]. A study 1 year after inpatient treatment of SAM showed that surviving children remained stunted [19]. These findings are important as there is a well-established positive correlation between height-for-age and development [1, 4, 20, 21]. Therefore, strategies to mitigate the negative impacts on child development and nutritional outcomes following inpatient treatment are needed in this vulnerable population.

A systematic review evaluating the evidence of psychosocial stimulation interventions for children with SAM indicated that based on two published studies in this population, there are positive impacts of this type of intervention on child development [22–24]. This review also clearly identified the need for further research evaluating feasible interventions incorporating psychosocial stimulation [22]. Furthermore, there are recommendations for psychosocial stimulation within NRU settings, but it is not clear that these interventions are being provided effectively [13, 22, 25]. There is also still a limited understanding of how best to implement behavior change interventions of nutrition and feeding and how effective such programs could be at improving developmental and nutritional outcomes particularly in children with SAM who may benefit most [5, 26, 27]. Finally, in children with SAM and acute illnesses, water, sanitation, and hygiene (WASH) becomes an important piece related to developmental and nutritional outcomes [28, 29]. However, nutrition interventions incorporating WASH for children with SAM are scarce at the present time [5, 28].

The Kusamala Program is an interactive counseling program that was designed by researchers, nurses, and clinicians for primary caregivers of children with SAM with the goal of improving developmental and nutritional outcomes [30]. It is an integrated intervention including three modules created from existing materials from the World Health Organization (WHO) and United Nations Children’s Fund (UNICEF): psychosocial stimulation, nutrition and feeding, and WASH [31–33]. The program has four sessions, one focused on each module followed by a summary session.

In order to quantitatively assess the effectiveness of the Kusamala Program in children hospitalized with SAM, a protocol for a pragmatic cluster-randomized controlled trial was designed (NCT03072433) [30]. An internal pilot cluster-randomized controlled trial was embedded within the full trial including the first participants enrolled to the study. A participatory focus group discussion (FGD) and in-depth interviews (IDIs) were done with hospital staff members to understand perceptions around the implementation of the Kusamala Program in a real NRU setting.

The objectives of this mixed methods feasibility study were:

1. To determine engagement and adherence rates of participants to the Kusamala Program
2. To obtain data of developmental outcomes of children after hospitalization with SAM to re-estimate the sample size for the full cluster-randomized controlled trial
3. To gain insight about potential barriers and enablers to implementation of the Kusamala Program

## Methods

This mixed methods feasibility study was done at the Moyo NRU at the Queen Elizabeth Central Hospital in Blantyre, Malawi. The internal pilot trial followed the same parallel design as the full cluster-randomized controlled trial and included the first 30 trial participants, which is standard for pilot studies [30, 34, 35].

The Good Reporting of A Mixed Methods Study framework was followed throughout this paper, in addition to the Consolidated Standards of Reporting Trials (CONSORT) 2010 statement with extension to randomized pilot trials (Additional file 1) and the Consolidated Criteria for Reporting Qualitative Research for the reporting of the qualitative component [36–38].

### Participants and informants

**Fig. 1:**
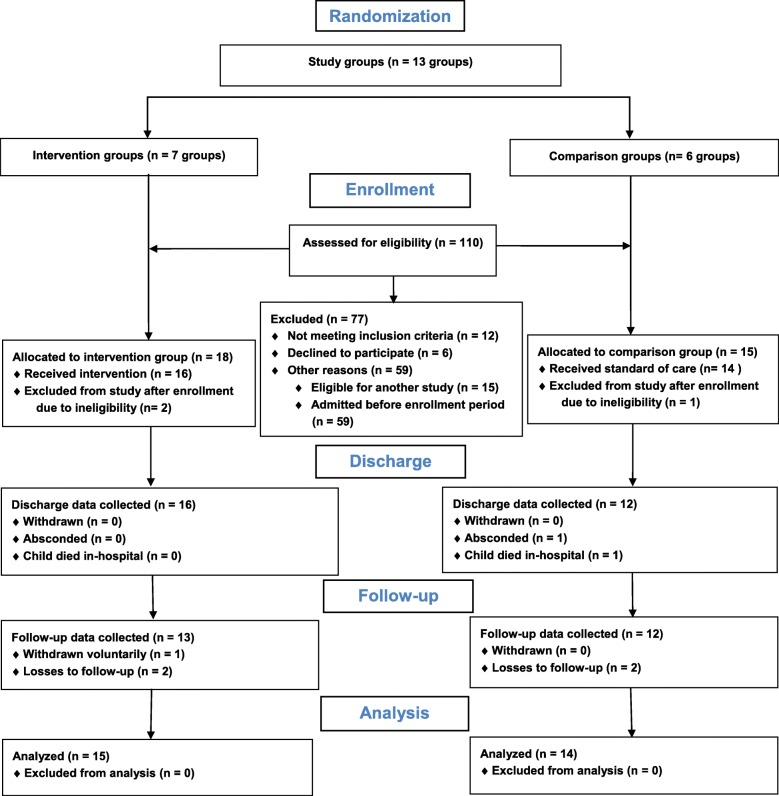
Modified version of CONSORT 2010 flow diagram of participants in the internal pilot trial

Primary caregivers and children were enrolled to the internal pilot trial between November 2016 and April 2017. Children with SAM and their primary caregivers at the NRU were screened for eligibility to participate by two staff members who were blinded to the allocation before and after enrollment. Between one and six primary caregivers and their children were assigned to each cluster. These clusters of participants were subsequently randomized to receive either the intervention or the standard of care according to a computer-generated random allocation sequence generated a priori by a biostatistician. Randomization of clusters was done on a particular day each week on a rolling basis. A study flow diagram summarizes the enrollment process and numbers according to the CONSORT 2010 Statement (Fig. 1) [39, 40].

Inclusion criteria:

1. Child 6–59 months of age with SAM
2. Child admitted to hospital because of SAM with medical complications per WHO guidelines [9]
3. Primary caregiver (self-identified) present at hospital

Exclusion criteria:

1. Primary caregiver declined to give informed consent
2. Child with a known terminal illness
3. Child requiring a surgical procedure

Primary caregivers gave written informed consent on behalf of themselves and their children. For primary caregivers who were illiterate or unable to write, a thumbprint was taken in the presence of an impartial witness. Pre-defined detailed information about the qualitative study was provided to potential informants, and they were given time to make an informed decision. Written or verbal records of the consent were obtained before the FGD and IDIs took place. All potential informants were made aware that participation is voluntary.

IDIs were conducted in June and July 2017, following the completion of recruitment of the internal pilot trial and once all children in the internal pilot trial were discharged from hospital. The FGD was completed in February 2018 after NRU nurses had been delivering the Kusamala Program for more than 1 year giving them enough experience to provide detailed information on delivery, implementation, and sustainability. The training provided to these nurses for the Kusamala Program has been presented in the study protocol [30]. Action research theory was applied within the FGD in which those who were actively conducting the Kusamala Program decided on the processes for conducting this qualitative research [41]. This enabled engagement and understanding of what these personnel felt were important issues with regard to the implementation of the Kusamala Program [41].

The IDIs followed a purposive sampling approach in which NRU staff members, including two field workers, one ward clerk, and two patient attendants, and the two core nurses at a neighboring pediatric ward at the Queen Elizabeth Central Hospital were identified. No eligible informants declined to participate. The FGD included the five permanent NRU nurses, all of whom were trained to conduct the Kusamala Program. One of the NRU nurses participated as both an informant and a leader of the FGD to encourage discussion with NRU nurses. Two local female enumerators fluent in English and Chichewa were also involved in completing, transcribing, and translating the FGD and IDIs, respectively. Both had prior experience in qualitative research and were individuals that informants had a trusting relationship with, allowing for credibility of the data. Informants were advised that enumerators’ goals were to confidentially obtain positive and negative information and that varying opinions were expected and encouraged. They were also notified that information that they shared would not impact their employment in any way. The enumerators kept notes during the data collection process to reflect on whether there were any potential biases that could influence the relationships with informants.

### Intervention and comparison

A computer-generated randomization scheme for each cluster at a ratio of one intervention week to one comparison week was used as described before. For the internal pilot trial, the ratio was not pre-specified as it simply included the first randomized clusters from the full trial [30].

Nurses facilitated the Kusamala Program in the play room in back bay of the NRU. This included four sessions over 4 days, beginning at around 3:15 PM, each involving 45 min of counseling of primary caregivers during which nurses provided information and facilitated discussions, followed by 45 min of interactive play supervised by nurses. Content of the Kusamala Program is described in further detail in the study protocol [30]. Primary caregivers were given take-home images following each of the sessions on nutrition and feeding and WASH, and a choice of Western or local toys was given to children following the psychosocial stimulation sessions. Upon completion of the Kusamala Program, primary caregivers were given certificates with their and their children’s names.

Aside from medical and nutritional therapy at the NRU, the standard of care included access to the play room in the NRU during weekday afternoons. Nurses told primary caregivers in the comparison clusters to use the same area where toys are available, but nurses did not facilitate interactive play sessions. This also acted as a means of blinding study personnel and participants to intervention delivery. When children were discharged from hospital, nurses or other NRU staff members counseled primary caregivers on basic nutrition and feeding and WASH messages as is standard practice. No further follow-up counseling was done as a component of this trial.

### Participant data and implementation outcomes

Data was collected upon enrollment, at discharge from hospital, and 6 months following discharge at the homes of participants. Baseline data collected included household information and primary caregiver characteristics as well as child characteristics including nutritional status assessed by anthropometry. Anthropometric measures included bilateral pitting edema, mid-upper arm circumference, weight-for-length or weight-for-height *z*-scores, length- or height-for-age *z*-scores, and weight-for-age *z*-scores per WHO standards [9, 42].

The primary outcome measure for the full cluster-randomized controlled trial is the MDAT [18]. This developmental assessment tool was specifically designed and standardized for assessing children up to 6 years of age in rural African contexts [18]. It contains 36 items of increasing difficulty in each of four developmental domains: gross motor, fine motor, language, and social development [18]. The MDAT was used at hospital discharge and follow-up 6 months later.

Participant engagement was measured by the proportion of primary caregivers enrolled to the intervention clusters who attended day 1 of the Kusamala Program; participant adherence was the proportion who attended all 4 days of the program and received a certificate of completion.

### FGD and IDI process

A semi-structured guide was created for the FGD and first included discussion questions followed by participatory activities. The first activity was problem identification and solving, in which informants, all of whom were nurses involved in delivering the Kusamala Program, were asked to identify actual or hypothetical problems around implementing the Kusamala Program. Another activity was to invite informants to each recall one issue, success, or unique moment encountered while delivering the Kusamala Program. The final activity was for each informant to imagine going to another NRU and convincing others to introduce the Kusamala Program. This FGD took place over two 3-h sessions in a private room led by one enumerator and the assigned NRU nurse, with support from a research assistant who audio-recorded the FGD and took detailed notes. The FGD was conducted in English since all NRU nurses are proficient and was transcribed verbatim.

An IDI guide with open-ended questions was designed and pilot-tested with a Chichewa-speaking staff member working in the NRU. The interview guide was designed to allow for the enumerator to use prompts and probes. Interviews were done one-on-one in a private setting in the NRU or an office nearby, allowing for informants to share information openly. The duration of each interview was between 15 and 30 min. Interviews were audio-recorded, transcribed verbatim, and translated back to English if done in Chichewa.

### Data management and analysis

Data for the internal pilot trial was double-entered into a Research Electronic Data Capture (REDCap) database and into WHO Anthro v3.2.2 [43, 44]. Discrepancies between double-entered data were resolved by referring to paper questionnaires. Data was subsequently analyzed in Stata 14 and WHO Anthro v3.2.2 for anthropometric *z*-score calculations [43, 45]. Descriptive statistics were done to summarize baseline and in-hospital characteristics of participants. Means and SDs were used for continuous variables; proportion of participants and percentages were used for categorical variables. MDAT *z*-scores standardized by age were calculated based on a reference population of children in Malawi [18]. The sample size for the full cluster-randomized controlled trial was recalculated based on mean MDAT *z*-scores of the four domains at follow-up.

NVivo 11 was used to analyze qualitative data from the FGD and IDIs [46]. Derivation of themes was data-driven, meaning that inductive codes arose from the qualitative data. These themes were categorized as being either barriers or enablers to implementation and sustainability of the Kusamala Program. Findings were shared with informants of the FGD and IDIs and checked for correctness to ensure accurate reporting.

### Ethical approval

Ethical approval for the cluster-randomized controlled trial was obtained from the University of Malawi College of Medicine Research and Ethics Committee and the Hospital for Sick Children Research Ethics Board. For the qualitative component, ethical approval was obtained from the University of Malawi College of Medicine Research and Ethics Committee and the Liverpool School of Tropical Medicine Research Ethics Committee.

## Results

### Internal pilot trial participants

A total of 13 clusters were included in the internal pilot trial to include the required first 30 participants enrolled to the trial. Of the 36 eligible primary caregivers, 30 were enrolled meaning that the acceptance rate was 83.3%. One primary caregiver in the intervention arm withdrew from the study.

### Baseline characteristics

Baseline characteristics and nutritional status of children are summarized in Table 1 and Table 2, while characteristics of primary caregivers and households are summarized in Table 3.

**Table 1:** Clinical characteristics of children admitted for inpatient treatment of SAM

Variables	All (*n* = 29)	Intervention (*n* = 15)	Comparison (*n* = 14)
Age (months)	18.4 ± 8.1	18.5 ± 8.5	18.3 ± 7.8
Sex female (%)	15/29 (51.7)	7/15 (46.7)	8/14 (57.1)
Neurodisability (%)	1/29 (3.4)	1/15 (6.7)	0/14 (0)
HIV status (%)			
Reactive	8/29 (27.6)	4/15 (26.7)	4/14 (28.6)
Non-reactive	19/29 (65.5)	11/15 (73.3)	8/14 (57.1)
Unknown	2/29 (6.9)	0/15 (0)	2/14 (14.3)
Previous inpatient admission (%)	9/29 (31.0)	6/15 (40.0)	3/14 (21.4)
Inpatient death (%)	1/29 (3.4)	0/15 (0)	1/14 (7.1)
Duration of hospital stay (days)^a^	7.9 ± 6.9	9.4 ± 8.5	6.1 ± 3.8

Values are presented as proportions (%) for categorical data or means ± SDs for continuous data

*HIV* human immunodeficiency virus, *SAM* severe acute malnutrition, *SD* standard deviation

^a^Excluding children who died in-hospital

**Table 2:** Baseline anthropometric indices of children admitted for inpatient treatment of SAM

Variables	All (*n* = 29)	Intervention (*n* = 15)	Comparison (*n* = 14)
Edema (%)			
Enrollment	7/29 (24.1)	3/15 (20.0)	4/14 (28.6)
Discharge	1/27 (3.7)	0/15 (0)	1/12 (8.3)
MUAC (cm)^a^			
Enrollment	11.3 ± 1.0	11.2 ± 1.2	11.4 ± 0.7
Discharge	11.5 ± 0.9	11.4 ± 1.0	11.6 ± 0.9
WLZ or WHZ^a^			
Enrollment	− 3.5 ± 0.7	− 3.5 ± 0.7	− 3.6 ± 0.7
Discharge	− 2.9 ± 1.2	− 3.0 ± 0.7	− 2.9 ± 1.7
LAZ or HAZ			
Enrollment	− 3.1 ± 1.5	− 3.2 ± 1.0	− 3.0 ± 1.8
Discharge	− 3.2 ± 1.4	− 3.4 ± 1.0	− 3.0 ± 1.8
WAZ^a^			
Enrollment	− 4.2 ± 0.6	− 4.2 ± 0.7	− 4.2 ± 0.6
Discharge	− 3.8 ± 1.1	− 4.1 ± 0.7	− 3.6 ± 1.5

Values are presented as proportions (%) for categorical data or means ± SDs for continuous data

*HAZ* height-for-age *z*-score, *LAZ* length-for-age *z*-score, *MUAC* mid-upper arm circumference, *SAM* severe acute malnutrition, *SD* standard deviation, *WAZ* weight-for-age *z*-score, *WHZ* weight-for-height *z*-score, *WLZ* weight-for-length *z*-score

^a^Excluding children with edema

**Table 3:** Baseline characteristics of primary caregivers and households of children with SAM

Variables	All (*n* = 29)	Intervention (*n* = 15)	Comparison (*n* = 14)
Age (years)	27.4 ± 10.2	27.3 ± 7.9	23.4 ± 12.6
Relationship to child (%)			
Mother	27/29 (93.1)	14/15 (93.3)	13/14 (92.9)
Grandmother	2/29 (6.9)	1/15 (6.7)	1/14 (7.1)
Has ever attended school (%)	19/29 (65.5)	11/15 (73.3)	8/14 (57.1)
Household income last month (USD equivalent)	28.66 ± 40.41	34.30 ± 51.46	22.05 ± 22.30
Household area (%)			
Urban	16/29 (55.2)	8/15 (53.3)	8/14 (57.1)
Rural	13/29 (44.8)	7/15 (46.7)	6/14 (42.9)

Values are presented as proportions (%) for categorical data or means ± SDs for continuous data

*SAM* severe acute malnutrition, *USD* United States dollar

### Participant engagement and adherence

Participant engagement, defined as the proportion of primary caregivers enrolled to intervention clusters that attended day 1 of the Kusamala Program, was 15/15 (100%). Of these, 13/15 (86.7%) attended all 4 days. Two primary caregivers did not attend all 4 days, as their children were discharged prior to completion of the four Kusamala Program sessions. Therefore, 13/13 (100%) of participants remaining in-hospital for the course of the 4-day intervention were adherent.

### Child developmental outcomes

**Fig. 2:**
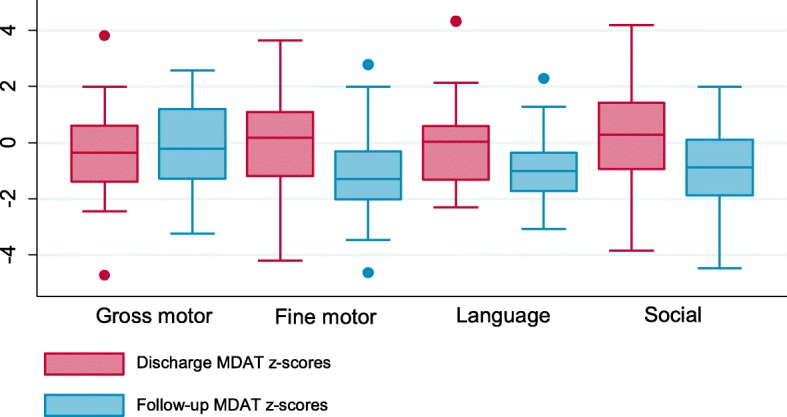
Boxplot of MDAT *z*-scores for gross motor, fine motor, language, and social domains in children with SAM at discharge and follow-up. Pooled MDAT *z*-scores for children in intervention and comparison arms. Excluding children with known neurodisabilities. MDAT Malawi Developmental Assessment Tool, SAM severe acute malnutrition

MDAT *z*-scores were computed for 12 children in comparison clusters and 14 in intervention clusters at discharge, and 11 children in each arm at follow-up. One child in a comparison cluster was excluded from the analysis of MDAT *z*-scores because of a pre-existing neurodisability. MDAT *z*-scores for gross motor, fine motor, language, and social domains at discharge and follow-up are displayed in Fig. 2.

#### Sample size recalculation

From the 22 children successfully followed up, the average MDAT *z*-score SD was calculated to be 1.55. An effect size of 0.5 was used to represent a potentially clinically significant change in MDAT *z*-scores to justify the implementation of the Kusamala Program. Cluster sizes from this internal pilot trial were small for the calculation of an intracluster correlation coefficient, and therefore a value of 0.05 was selected based on the postulation that developmental outcomes will not vary greatly by cluster. Using *α* of 0.05 and 80% power, an estimated minimum of 158 children per arm (*N* = 316) should be included in the analysis of the full trial [30]. This number is slightly lower than the original sample size calculation of 160 children per arm (*N* = 320) in the original study protocol, which will be followed since it is higher than the recalculated sample size [30]. To account for contingencies, an increase in the sample size by 25% would be necessary meaning that 200 children per arm (*N* = 400) should be included in the full trial.

### Barriers and facilitators to implementation

The three main barriers to implementation of the Kusamala Program included caregivers’ perceived value of the Kusamala Program, prioritization of other ward activities, and shortages of staff.

It was raised in two IDIs and in the FGD that some caregivers did not understand the purpose of the program before beginning the intervention and were therefore less willing to participate. This was often because caregivers were preoccupied by their child’s illnesses or conditions such as cerebral palsy. In the FGD, nurses discussed how to approach primary caregivers to ensure that they understand the Kusamala Program before making their decision to participate.

One of the children was a cerebral palsy patient. To the caregiver, the talk of play and stimulation was not applicable. I should bring the message in a different way that this caregiver should understand. After counselling, she understood, and you could tell with how she was playing with her child. The mother was understanding of how important it is compared to the perceptions she had at the beginning. (Nurse, FGD)

What I have seen is that some take the program as normal but for some because of their beliefs and misunderstanding of the program as well as religious beliefs, it is difficult to welcome the program, but others do welcome it well without any problem. (Field Worker, IDI)

The other barriers included prioritization of other ward activities, identified by two informants in IDIs, and shortages of staff, described by three participants in IDIs. Both of these barriers were also discussed by nurses in the FGD.

Especially when you are in the ward and you need to start the intervention, when you’d like to start to teach the caregivers, you have got other things to do and you don’t always start on time. (Nurse, FGD)

Acting as a potential solution to these barriers, four informants participating in IDIs felt that others working in the NRU aside from nurses could be involved in delivering the Kusamala Program. They explained that other staff members such as cleaners, patient attendants, or ward clerks could be trained to deliver the Kusamala Program and that this would allow for the nurses to conduct other work.

In these wards, there are those we call domestic staff, so it starts from cleaners, patient attendants, so such people are the ones that would do that as nurses are always busy and they cannot do that but the patient attendant, ward clerk, cleaners are the ones who can do this or help in this. (Ward Clerk, IDI)

When you look at the setting of the Moyo NRU itself it sometimes is not busy, sometimes is a very busy ward. So, let’s say there are three or four nurses who work on shifts. You can’t have all of them at the same time and the ward is busy. I think it would be very ideal to actually have someone outside of that. (Field Worker, IDI)

Nurses in the FGD and two IDIs recognized motivation and work ethic as an additional enabler of the Kusamala Program. Linked to this enabler was the inclusion of refresher training to maintain this motivation and increase knowledge, which was mentioned by nurses in the FGD and four informants that completed IDIs.

What made it to work so well is mainly from the fact that the ones who are involved in this program work very hard. (Field Worker, IDI)

Perhaps there is a need for refresher training more frequently so that people should be well updated and acquire new knowledge and skills to support the patients. (Field Worker, IDI)

## Discussion

This feasibility study applied a mixed methods approach, first with an internal pilot cluster-randomized controlled trial evaluating child development and implementation outcomes. Exploratory qualitative methods through a FGD and IDIs were then utilized to gain insight about potential barriers and enablers to implementation of the Kusamala Program from the perspectives of personnel involved in delivering the Kusamala Program.

A systematic review of studies evaluating interventions to improve child development showed that implementation outcomes are rarely described [3]. This is the first study to present implementation outcomes of an intervention that incorporates psychosocial stimulation for children with SAM [22]. The engagement and adherence rates to the Kusamala Program were 100% and 86.7%, respectively.

Child development was assessed using the MDAT, a culturally appropriate tool to evaluate four domains of development. MDAT *z*-scores from this internal pilot trial were used to recalculate the sample size for the full trial, as no other studies have examined follow-up MDAT *z*-scores of children after inpatient treatment of SAM. Because the recalculated sample size estimate was lower than the estimate in the protocol, the original estimate will be accepted [30, 34].

Adjustments to the full cluster-randomized controlled trial and the Kusamala Program were made according to results of this feasibility study. Based on the potential barrier of prioritization of other ward activities which could interfere with starting the sessions on time, a fidelity assessment of the Kusamala Program was added to the full trial. An enumerator trained in early child development will assess the delivery of 20% of intervention sessions with an observational assessment tool based on the WHO Care for Child Development Package and an overall rating of the delivery of each session using a Likert scale [33]. The enumerator will also provide feedback to nurses to improve delivery of the Kusamala Program. Furthermore, NRU nurses will receive refresher training twice per year since this was identified as an enabler to sustain effective delivery of the Kusamala Program.

Another addition was a 3-day training session for NRU nurses in early child development after completing recruitment of the internal pilot trial. Although all nurses have been previously trained on counseling components of the Kusamala Program, a more comprehensive overview of child development will better equip them to conduct play sessions. Child development training is becoming widely available in Malawi and other low- and middle-income countries through efforts such as UNICEF’s Early Child Development initiatives and therefore could be accessible for other NRU staff members [47, 48].

The Kusamala Program was also shortened to last 75 min per session, totaling 5 h per week, as it was impractical for NRU nurses to have 90-min sessions per week day in addition to their normal ward duties which are outlined in Additional file 2. The logistical constraint of starting at a given time was also a concern raised in the FGD due to other ward activities and shortages of staff. Conducting the Kusamala Program over 75 min each day may still be difficult in combination with normal ward duties, and therefore fidelity assessments will allow for evaluation of delivery of key messages within the intervention sessions. Solutions to this barrier were also brought forth whereby other NRU staff members aside from nurses could assist with the delivery of the Kusamala Program. Although this will not be done within the full trial, this could be evaluated and potentially implemented in practice.

### Limitations

One limitation of the internal pilot trial is that enrollment was lower than anticipated. This could be attributed to the scale-up of community-based efforts to manage malnutrition. Therefore, the average and median cluster sizes of 2.6 and 2.0 participants, respectively, were smaller than expected. This meant that an intracluster correlation coefficient was not calculated from the internal pilot study data for the recalculation of the sample size.

There were also few eligible informants for FGD and IDIs as there are limited core staff members, which is a characteristic of a low-resource setting. This meant that it was not possible to reach data saturation. However, the aim was to collect detailed information from those who are most familiar with the NRU and who are key to the implementation of interventions like the Kusamala Program in real NRU settings.

## Conclusions

The Kusamala Program for primary caregivers of children with SAM was evaluated in an NRU setting in Malawi using a mixed methods approach. High engagement and adherence of primary caregivers to the Kusamala Program was achieved within an internal pilot trial. The Kusamala Program is feasible to implement upon addressing barriers and strengthening enablers.

## Additional files

Additional file 1: CONSORT 2010 checklist for randomized pilot and feasibility trials. (DOCX 21 kb)

Additional file 2: Moyo NRU daily schedule and timing of the Kusamala Program. (DOCX 14 kb)

Additional file 3: The dataset supporting the conclusions of this article. (XLS 116 kb)

## Abbreviations

CMAM: Community-Based Management of Acute Malnutrition
CONSORT: Consolidated Standards of Reporting Trials
FGD: Focus group discussion
IDI: In-depth interview
MDAT: Malawi Developmental Assessment Tool
NRU: Nutritional rehabilitation unit
SAM: Severe acute malnutrition
SD: Standard deviation
UNICEF: United Nations Children’s Fund
WASH: Water, sanitation, and hygiene
WHO: World Health Organization
