# Supplementary material for: A mixed methods feasibility study of the Kusamala Program at a nutritional rehabilitation unit in Malawi
Source: Pilot Feasibility Stud. 2018 Sep 24;4:151. doi: 10.1186/s40814-018-0347-8 (PMC6151933; doi:10.1186/s40814-018-0347-8)
Supplement: Supplementary file 1 — CONSORT 2010 checklist for randomized pilot and feasibility trials. (DOCX 21 kb) [file 40814_2018_347_MOESM1_ESM.docx]

**Additional file 1.** CONSORT 2010 checklist for randomized pilot and feasibility trials.

| **Section/topic** | **#** | **Checklist item** | **Reported on page #** |
| --- | --- | --- | --- |
| **TITLE AND ABSTRACT** | | |  |
| Title | 1a | Identification as a randomised trial in the title. | N/A |
| Structured summary | 1b | Structured summary of pilot trial design, methods, results, and conclusions. | 3 |
| **INTRODUCTION** | | |  |
| Background and objectives | 2a | Scientific background and explanation of rationale for future definitive trial, and reasons for randomised pilot trial. | 5-7 |
|  | 2b | Specific objectives or research questions for pilot trial. | 7 |
| **METHODS** | | |  |
| Trial design | 3a | Description of pilot trial design (such as parallel, factorial) including allocation ratio. | 5-8 |
|  | 3b | Important changes to methods after pilot trial commencement (such as eligibility criteria), with reasons. | 20-22 |
| Participants | 4a | Eligibility criteria for participants. | 9-10 |
|  | 4b | Settings and locations where the data were collected. | 8-10 |

|  | 4c | How participants were identified and consented. | 9-10 |
| --- | --- | --- | --- |
| Interventions | 5 | The interventions for each group with sufficient details to allow replication, including how and when they were actually administered. | 10-11 |
| Outcomes | 6a | Completely defined prespecified assessments or measurements to address each pilot trial objective specified in 2b, including how and when they were assessed. | 11-12 |
|  | 6b | Any changes to pilot trial assessments or measurements after the pilot trial commenced, with reasons. | N/A |
| Sample size | 7 | Rationale for numbers in the pilot trial. | 8 |
| Randomization: |  |  |  |
| Sequence generation | 8a | Method used to generate the random allocation sequence. | 8 |
|  | 8b | Type of randomisation(s); details of any restriction (such as blocking and block size). | 8 |
| Allocation concealment mechanism | 9 | Mechanism used to implement the random allocation sequence (such as sequentially numbered containers), describing any steps taken to conceal the sequence until interventions were assigned. | 8 |
| Implementation | 10 | Who generated the random allocation sequence, enrolled participants, and assigned participants to interventions. | 8-9 |
| Blinding | 11 | If done, who was blinded after assignment to interventions (eg, participants, care providers, those assessing outcomes) and how. | 11 |
| Analytical methods | 12 | Methods used to address each pilot trial objective whether qualitative or quantitative. | 13 |

| **RESULTS** | | |  |
| --- | --- | --- | --- |
| Participant flow | 13a | For each group, the numbers of participants who were approached and/or assessed for eligibility, randomly assigned, received intended treatment, and were assessed for each objective. | 10,14 |
|  | 13b | For each group, losses and exclusions after randomization, together with reasons. | 14 |
| Recruitment | 14a | Dates defining the periods of recruitment and follow-up. | 8,9-10 |
| Baseline data | 15 | A table showing baseline demographic and clinical characteristics for each group. | 14-17 |
|  | 16 | For each objective, number of participants (denominator) included in each analysis. If relevant, these numbers should be by randomised group. | 14-17 |
| Outcomes and estimation | 17 | For each objective, results including expressions of uncertainty (such as 95% confidence interval) for any estimates. If relevant, these results should be by randomised group . | 14-17 |
| Ancillary analyses | 18 | Results of any other analyses performed that could be used to inform the future definitive trial. | 17-18 |
| Harms | 19 | All important harms or unintended effects in each group. | N/A |
| **DISCUSSION** | | | |
| Limitations | 20 | Pilot trial limitations, addressing sources of potential bias and remaining uncertainty about feasibility. | 23 |
| Generalizability | 21 | Generalisability (applicability) of pilot trial methods and findings to future definitive trial and other studies. | 20-23 |

| Interpretation | 22a | Interpretation consistent with pilot trial objectives and findings, balancing potential benefits and harms, and considering other relevant evidence. | 20-23 |
| --- | --- | --- | --- |
|  | 22b | Implications for progression from pilot to future definitive trial, including any proposed amendments. | 20-23 |
| **OTHER INFORMATION** |  |  |  |
| Registration | 23 | Registration number for pilot trial and name of trial registry. | 3 |
| Protocol | 24 | Where the pilot trial protocol can be accessed, if available. | 7 |
| Funding | 25 | Sources of funding and other support, role of funders. | 26 |
|  | 26 | Ethical approval or approval by research review committee, confirmed with reference number. | 13-14,26 |
