# Supplementary material for: A mixed methods feasibility study of the Kusamala Program at a nutritional rehabilitation unit in Malawi
Source: Pilot Feasibility Stud. 2018 Sep 24;4:151. doi: 10.1186/s40814-018-0347-8 (PMC6151933; doi:10.1186/s40814-018-0347-8)
Supplement: Supplementary file 2 — Moyo NRU daily schedule and timing of the Kusamala Program. (DOCX 14 kb) [file 40814_2018_347_MOESM2_ESM.docx]

**Table S3** Moyo NRU daily schedule and timing of the Kusamala Program.

| **Time** | **Activity** |
| --- | --- |
| 7:30AM | Nurses begin their shifts |
| 7:30AM – 8:00AM | Nurses’ handover |
| 8:00AM – 9:00AM | Nurses tend to patients and clean beds |
| 9:00AM | *Feeds* |
| 9:15AM – 11:00PM | Ward rounds with nurses and clinicians |
| 12:00PM | *Feeds* |
| 12:15PM – 1:30PM | Nurses’ lunch break |
| 1:30PM – 2:00PM | Nurses tend to patients and administer medication |
| 2:00PM – 3:00PM | Ward rounds with nurses and clinicians |
| 3:00PM | *Feeds* |
| **3:15PM – 4:30PM** | **Kusamala Program** |
| 4:30PM | Nurses finish their shifts |
